# Does subjective life expectancy matter in purchasing life insurance among middle-aged and older adult? Evidence from China

**DOI:** 10.3389/fpubh.2024.1426366

**Published:** 2024-09-12

**Authors:** Xuejiao Chen, Yunhan Guo, Chang Lu, Yizhen Wang, Hanshuo Wen

**Affiliations:** School of Economics, Minzu University of China, Beijing, China

**Keywords:** subjective life expectancy, life insurance participation, Chinese middle-aged and older adult, China Health and Retirement Longitudinal Study, Probit model, Tobit model

## Abstract

Population-wide increase in life expectancy is a source of aggregate longevity risk. Life insurance is a natural instrument to manage the risk. Previous studies used chronological age to examine the relationship between aging and life insurance purchase, which ignored the impact of subjective life expectancy-the real perception of remaining time. Therefore, this study aims to fill the lack in this area and to explore in depth the relationship between subjective life expectancy and purchasing life insurance among middle-aged and older adult at micro perspective. This paper utilizes data from the China Health and Retirement Longitudinal Study (CHARLS) over a period of 4 years to construct both Probit and Tobit models. The findings reveal that subjective life expectancy positively affects the likelihood of participation and the extent of life insurance among the middle-aged and older adult population in China. IV model estimation results show good robustness of the results. Meanwhile, there is also heterogeneity in the effect with respect to gender, hukou, education and wealth. The findings provide new perspective to explain the subjective motivation of purchasing life insurance in China.

## 1 Introduction

The average life expectancy grows rapidly from 68.6 years in 1990 to 78.1 years in 2023, an increase of nearly 10 years. According to international standards, China has become an aging society. The rapid aging of the population presents great challenges for the individual in terms of risk management, especially in economic. In addition, the pension insurance replacement rate continues to decline, which has intensified the economic risk and posed a challenge to middle-aged and older adult of our country. A key dimension to deal with the personal financial risks is the ability to absorb financial shocks associated with increased lifespan. By purchasing life insurance, middle-aged and older adult get protection for their future and ensure that their families or children are going to receive insurance benefit in the event of their incapacity to work or death, thus alleviating their burden (1). In addition to reducing financial pressure, purchasing life insurance improves their quality of life and happiness, and maximizes their utility (2–6). To this end, from a financing perspective, purchasing life insurance provides protection to the individual and reduces exposure to perils.

Life insurance is an important financial tool in later life that has received a large amount of attention over the last several decades (7–9). However, even to this day, risk management of residents’ is mainly focused on the social insurance, and the life insurance is seriously lagging. Previous studies have shown that macro-environmental and micro-personal factors that affect purchasing life insurance, including income, wealth, saving rate, age, gender, marital status, the number of children and so on (10–13). Although there is a large body of research identifying factors of purchasing life insurance, surprisingly little attention has been given to the subjective feeling. Behavioral finance refers to the fact that people tend to be imperfectly rational in their financial decisions, and subjective feeling is an intrinsic driver of behavior (14, 68, 69). Chui and Kwok (15) found that individualism has a positive effect on life insurance purchases. Laura et al. (16) explored the effect of intentions and behavior on the purchase of life insurance and private pensions without considering expectations. Park et al. (17) found that highly anxious people used savings/wealth accumulation as the only alternative to insurance. Nomi and Sabbir (18) found that attitudes, subjective norms, risk aversion motives, and saving motives all have significant positive effect on consumers’ willingness to purchase life insurance. Subjective life expectancy is the most important subjective feeling of the individual because it impacts individuals’ decisions on how to spend their remaining time (19, 20).

Subjective life expectancy is an individual’s subjective feel, perception and prediction to think how long can a person live (21). When families aim to maximize utility over the life cycle under uncertainty, the optimism of family members’ expectations of the future may affect the allocation of family financial assets due to the obvious differences in the risk, return, and allocation of different types of financial assets such as saving, stock, and insurance (22, 23). For individuals, subjective perceptions of life expectancy are critical to their retirement plans, health insurance needs, and consumption behaviors, and in a broader sense may play a role in any choice related to maximize an individual’s expected benefits (24, 25). Personal misunderstanding of survival probability is a key factor leading to low annuity demand, and governments need to use policy intervention to raise awareness of life expectancy and help individuals make more reasonable retirement planning and annuity purchase decisions (26, 27). However, to date, the research connecting purchasing life insurance to subjective life expectancy is still underdeveloped (2). This paper aims to fill the gap, and study the impact of subjective life expectancy on purchasing life insurance.

As for life insurance, subjective life expectancy might provide an important source of information over and above population actuarial estimates. As triggering time in getting life insurance benefit is a function of initial time and age of death, subjective life expectancy provides the individual with his/her own unique time frame to guide decision-making (28). Doerr and Schulte (29) based on data from the German Savings and Pension Insurance Survey in 2005 found that subjective life expectancy significantly and positively influences the likelihood of participating pension insurance. Bucher-Koenen and Kluth (30) studied the German private life insurance market and obtain similar findings which subjective life expectancy has greater impact on women than men. Rappange et al. (31) argued that when subjective expectation is inaccurate (e.g., overly pessimistic), it may lead to non-optimal decisions. Mittal et al. (32), showed that subjective life expectancy is crucial for individuals and can influence many of their decisions, such as long-term care insurance purchase.

There are differences in the findings of articles that have been published on the relationship between subjective life expectancy and the willingness to purchase life insurance. Zerriaa et al. (33) investigated factors influencing the demand for life insurance in Tunisia and find that life expectancy at birth stimulates the demand for life insurance. Kabrt (34) in studying the Visegrád Group (V4) by using the OECD data, also obtained a positive correlation between the demand for life insurance and life expectancy. However, Donghui Li et al. (35), also studying the determinants of life insurance in the OECD, argued that the demand for life insurance decreases with life expectancy. Browne and Kim (36) argued that average life expectancy reflects the probability of death, and that longer life expectancy implies a lower probability of premature death. Therefore, life expectancy is negatively related to the demand for life insurance. In addition, other scholars (37, 38) argued that the two are irrelevant and ambiguous. How does subjective life expectancy affect the purchase of life insurance for Chinese? To what extent is purchasing life insurance influenced by subjective life expectancy among middle-aged and older adult in China? With the increase of aging population, it has highly theoretical value and strong reality significance to answer this question.

Based on the above analysis, this paper makes contributions as follows. It adds to the existing literature by providing further insights into subjective life expectancy, which is one of the important subjective determinants in life insurance demand. The finding provides new micro evidence on purchasing life insurance among middle-aged and older adult in China. And it also expands the research in decision-making from subjective feeling perspective.

The specific paragraph structure of the paper is organized as follows: after this introduction, section 2 presents the analysis and hypotheses about the mechanisms of the paper, section 3 describes the data sources and the variables included in the analysis. Section 4 presents the empirical analysis, and Section 5 concludes with the main conclusions and recommendations. Section 6 is discussion.

## 2 Theoretical background

Yaari (39) proposed the demand theory of life insurance, which emphasizes that people purchase life insurance to mitigate or eliminate the consequences of uncertainty about future income cash flows, so as to maximize lifetime consumption utility. Subjective life expectancy is a quantification of an individual’s perceived remaining life expectancy, and the result can represent the probability that an individual expects to live to a certain age. When the subjective life expectancy of middle-aged and older adults increases, they may be more concerned about the stability and sustainability of future income cash flows. As a result, to cope with future uncertainty, middle-aged and older adults may increase their demand for life insurance. In addition, according to the life-cycle hypothesis, in order to maximize utility over their life cycle, consumers tend to consume less and save more during the career stage to ensure that they can maintain the same level of consumption during the retirement stage and achieve a smooth transition of utility. When middle-aged and older people have more optimistic subjective expectations of their life expectancy, it means that they expect to live longer in old age, which may lead to a greater tendency to hold life insurance in order to obtain more insurance benefits.

A higher subjective life expectancy may also change people’s perception of risk (40). As life expectancy increases, people become more concerned about longevity risk (41, 42), which means the financial stress associated with living longer than expected. In response to this risk, people may be inclined to purchase more life insurance products to ensure that they can live comfortably even if they live a long time. This leads to the following hypothesis:

*H1*: The extension of subjective life expectancy has a significant positive impact on middle-aged participation in life insurance.

Behavioral decision theory emphasizes the cognitive limitations of decision-makers in identifying problems and choosing solutions. Concerning the need for life insurance, individuals may have differences in assessing subjective life expectancy due to differences in cognitive ability (43). Individuals with higher cognitive abilities may be better able to accurately assess their own life expectancy (44) and make more rational insurance purchasing decisions accordingly. Second, risk preference is also an important factor influencing the demand for life insurance. Individuals have different levels of risk preferences, as well as differences in their perception and tolerance of risk (45). At the same time, individuals with higher risk preferences may be more willing to hedge against possible future risks by purchasing life insurance, while individuals with lower risk preferences may have a lower demand for life insurance. As individuals are influenced by multiple factors such as cognitive ability and risk preference in the decision-making process, it leads to differences in their needs for life insurance. Therefore, we believe that middle-aged and old-aged groups with different age, education level, health level and other characteristics are likely to make different life insurance purchase decisions even based on the same subjective life expectancy. This paper further proposes the following hypotheses:

*H2*: There is heterogeneity in the impact of subjective life expectancy on life insurance participation.

## 3 Materials and methods

### 3.1 Data and sample

This paper uses four waves of data from the 2011, 2013, 2015, and 2018 China Health and Retirement Longitudinal Study (CHARLS). CHARLS is a national survey designed to provide comprehensive and high-quality data. CHARLS database focuses on providing information on the personal characteristics, household structure, health status, healthcare, insurance, work, retirement, pension, income, and assets of people aged 45 and older in China. Samples were drawn in four stages: county sampling, village/household sampling, household sampling, and individual sampling. The national baseline survey was conducted in 2011–12, Wave 2 in 2013, Wave 3 in 2015, and Wave 4 in 2018. The baseline survey covered 17,708 individuals in 28 provinces, 150 districts and 10,257 households across the country. Samples covered households in different parts of the country and from different socio-economic backgrounds. Follow-up surveys included return visits to the baseline survey sample and interviews with new entrants. As a longitudinal follow-up survey program, CHARLS captures changes in individual behaviors and attitudes over time. In this paper, middle-aged and older people aged over 45 are selected as the target sample, and a total of 10,611 observations are obtained after removing missing data on key variables.

### 3.2 Variables

There are two dependent variables in this study. One of them is whether or not an individual participates in life insurance, which is a binary variable based on the CHARLS questionnaire question “Do you currently receive or expect to receive or contribute to any life insurance?” The answer “Yes” is defined as “having life insurance” and assigned a value of “1.” And the answer “No” is defined as “do not have life insurance” and assigned a value of “0.”Another dependent variable is the premium expenditure for life insurance. According to the CHARLS questionnaire question, “How much is the payment amount?” The answers are uniformly converted into yuan/year. In our study, life insurance mainly refers to the traditional death insurance, the insurance product that pays the insurance benefits to the beneficiary at the death of the insured, excluding the annuity.

The core independent variable is subjective life expectancy, which is measured in this paper using the subjective probability of survival. The CHARLS questionnaire sets different target ages depending on the age of the respondent (75 for respondents under 65 years old, 80 for respondents between 66 and 69 years old, 85 for those between 70 and 74 years old, and so on), and then respondents are asked how likely it is that they would live to the target age, with the response options being “Almost impossible,” “Not very likely,” “Maybe”, “Very likely,” “Almost certain,” respectively, assigned a value of 1–5. The higher the value, the more optimistic the sample are about their life expectancy. We assign answers 1 through 5 to correspond to 0, 25, 50, 75, and 100%, respectively (46).

Control variables include respondents’ age, gender, hukou, education level, marital status, physical status, participation in public pension insurance, number of surviving children, housing loan and household assets. These are variables that may affect the subjective life expectancy and participation in life insurance of the respondents. Among them, public pension insurance crowds out the demand for life insurance among middle-aged and older adult (47). The number of children contributes to the demand for life insurance among middle-aged and older adult from the perspective of bequest motives (48, 49). The detailed definitions of the variables in this paper are presented in Table 1.

**Table 1:** Definition of variables.

Variables		Symbol	Definition
Dependent variable	Participation of life insurance	LI	Whether the respondent has life insurance (yes = 1; no = 0)
	Premium expenditure for life insurance	Prem	Total expenses on life insurance premiums (logarithm)
Key variable	Subjective life expectancy	SLE	Individual’s subjective life expectancy (equal to the subjective probability of survival answered by the respondent in the questionnaire)
Control variables	Age	Age	Age of respondents(logarithm)
	Gender	Gender	Gender of respondents (male = 1; female = 0)
	Hukou	Hukou	Hukou type of respondents (urban = 1; rural = 0)
	Education	Edu	Education level of respondents (primary school or below = 1; junior high school = 2; senior high school = 3; college or above = 4)
	Spouse	Spouse	Whether the respondent has a spouse living with him/her (yes = 1; no = 0)
	Physically status	Health	Physical level of respondents (very good = 1; good = 2; fair = 3; poor = 4; very poor = 5)
	Public pension insurance	PPI	Whether the respondents have public pension insurance (yes = 1; no = 0)
	Num of Children	Nchild	Number of surviving children
	Housing loan	Loan	Whether take mortage to purchase houses now (yes = 1; no = 0)
	Asset	Asset	Total household assets of respondents (logarithm)

### 3.3 Model setting

The Probit model is used to test the effect of subjective life expectancy among middle-aged and older adult on their participation in life insurance. The regression equation is as follows:


(1) $$\mathrm{Pr}\left(LI=1|SLE,X\right)=\Phi \left(\alpha +\beta_{1}SLE+\beta_{2}X+\varepsilon \right)$$

Where LI represents the participation in life insurance of individual. SLE represents the subjective life expectancy of individual, as measured by the subjective probability of survival as answered by the respondents in the questionnaire. X represents a set of individual level control variables, including age, gender, hukou, education, spouse, physical status, participation in public pension insurance, number of surviving children, housing loan and household assets. ε is a random disturbance term.

Then, we measure life insurance participation by premium spending. Since the household premium expenditure without life insurance is zero, which is censored, the effect of subjective life expectancy on the level of life insurance participation was estimated using the following Tobit model:


(2) $$y^{*}=\alpha +\beta_{1}SLE+\beta_{2}X+\varepsilon ,Y=\max\left(0,y^{*}\right)$$

In Equation 2, Y represents the degree of participation in life insurance, measured by premium expenditure; other control variables are the same as in Equation 1.

## 4 Results

### 4.1 Descriptive statistical analysis

In order to reduce the difference between the magnitude of the data variables and avoid the impact of heteroscedasticity on the regression results, the variable premium expenditure and household assets are logarithmized in this paper. From the descriptive statistics table (Table 2), it can be seen that the sample size is 10,611, and the mean value of life insurance participation is 0.038, with a minimum value of 0 and a maximum value of 1, indicating that the number of people who have not purchased life insurance is high. The mean value of subjective life expectancy is 0.509, indicating that the subjective life expectancy of the middle-aged and older adult is more optimistic. The standard deviation is 0.318, indicating subjective differences in the estimation of life expectancy between individuals.

**Table 2:** Results of descriptive statistics.

Variable	Obs	Mean	Std.Dev.	Min	Max
LI	10,611	0.038	0.191	0	1
Prem	10,611	0.263	1.352	0	10.82
SLE	10,611	0.509	0.318	0	1
Age	10,611	60.433	8.587	45	111
Gender	10,611	0.502	0.5	0	1
Hukou	10,611	0.193	0.394	0	1
Edu	10,611	1.44	0.715	1	4
Spouse	10,611	0.841	0.365	0	1
PPI	10,611	0.813	0.39	0	1
Nchild	10,611	2.838	1.392	0	10
Asset	10,611	8.718	1.59	1.609	16.656
Loan	10,611	0.029	0.168	0	1
Health	10,611	3.369	1.052	1	5

### 4.2 Benchmark regression results

Table 3 reports the benchmark regression results of the impact of subjective life expectancy on life insurance purchases for middle-aged and older adult. Among them, Column (1) and (2) are listed as the estimation results of the Probit model by gradually adding the control variables. Column (3) reports the average marginal effect of the Probit model. Column (4) and (5) are the estimation results of Tobit model with control variables. The results showed that the effect of subjective life expectancy on life insurance was significantly positive regardless of whether the control variable was included. This result validates the H1 hypothesis, indicating that increasing subjective life expectancy has a significant positive impact on life insurance purchase intention for middle-aged and older people. Specifically, the results of Column (2) show that the coefficient of subjective life expectancy of the explanatory variables is 0.189, which is significant at the 5% level, indicating that subjective life expectancy can promote the demand for life insurance for middle-aged and older adult. The results of Column (3) shows that when subjective life expectancy increases by one unit, the probability of middle-aged and older adult buying life insurance increases by 0.015 percentage points. The results of Column (5) show that the coefficient of subjective life expectancy of the explanatory variable was 0.116, which was significant at the 1% level, indicating that the increase in subjective life expectancy has a significant positive impact on the premium expenditure of life insurance.

**Table 3:** Benchmark regression results.

	(1)	(2)	(3)	(4)	(5)
Variables	LI	LI	Margins	Prem	Prem
SLE	0.340***	0.189**	0.015**	0.205***	0.116***
	(0.072)	(0.081)	(0.006)	(0.041)	(0.043)
Age		−0.018***	−0.001***		−0.008***
		(0.004)	(0.000)		(0.002)
Gender		−0.076	−0.006		−0.058**
		(0.049)	(0.004)		(0.027)
Hukou		0.009	0.001		0.007
		(0.064)	(0.005)		(0.036)
Edu		0.229***	0.018***		0.179***
		(0.032)	(0.003)		(0.020)
Spouse		−0.008	−0.001		−0.011
		(0.078)	(0.006)		(0.038)
PPI		0.160**	0.012**		0.088***
		(0.063)	(0.005)		(0.034)
Nchild		−0.061***	−0.005***		−0.025**
		(0.023)	(0.002)		(0.011)
Asset		0.084***	0.007***		0.043***
		(0.016)	(0.001)		(0.009)
Loan		0.034	0.003		0.006
		(0.126)	(0.010)		(0.078)
Health		0.017	0.001		0.007
		(0.024)	(0.002)		(0.013)
Constant	−1.959***	−1.910***		0.158***	0.076
	(0.046)	(0.298)		(0.025)	(0.160)
Observations	10,611	10,611	10,611	10,611	10,611

Standard errors in parentheses.

***p < 0.01, **p < 0.05, *p < 0.1.

### 4.3 Endogeneity analysis

In the regression analysis of subjective life expectancy on the demand for life insurance, there is a possibility of endogeneity in the regression results due to the effect of omitted variables and mutual causality. Therefore, Instrumental Variables (IV) is used to analyze the regression for endogeneity. The existing literature (50–52) mostly uses whether parents live long as an instrumental variable (Parents). The reason for choosing is that studies have demonstrated that an individual’s subjective life expectancy is influenced by the longevity of relatives (53). By observing the age of their parents, respondents form and revise their subjective expectations of their own longevity. The longer the parents live, the longer the individual’s subjective life expectancy. Parental longevity is closely related to the independent variable of an individual’s subjective life expectancy. Concurrently, the longevity of one’s parents does not influence the dependent variable, which is the individual’s behavior in purchasing life insurance. Therefore whether parents live long is a valid instrumental variable. In this paper, the definition of whether parents live long is as follows: if parents are alive, they are considered to be long-lived parents. Second, the probability of survival is low considering that some samples are close to 60 years old and the actual age of parents is over 80 years old. Ultimately, this paper defines samples whose parents are actually surviving at an age of 80 or more as having long-lived parents.

Table 4 shows the regression results of the effect of subjective life expectancy on the likelihood of life insurance participation and the participation degree after the inclusion of the instrumental variable. The bottom reports the results of the Wald tests for subjective life expectancy endogeneity, which reject the hypothesis that there is no endogeneity of the variable at the 5% level, that is, there is an endogeneity of the independent variable subjective life expectancy. To further test the correlation, subjective life expectancy was included as the explained variable and regression was conducted using instrumental and other control variables as explanatory variables. The regression results showed that the instrumental variables were positively associated with the core explanatory variables at the 1% significance level, indicating that the instrumental variables satisfied the correlation with the endogenous variables. Secondly, it is necessary to pay attention to the effectiveness of the instrumental variables. From the regression results, the stage *F* value of the instrumental variables is 31.6354 (>10) (54), indicating that there is no weak instrumental variable problem in using whether parents live or long as the instrumental variable. The results of the second stage showed that the regression coefficient of subjective life expectancy was still significantly positive. This shows that subjective life expectancy still has a significant positive impact on the participation of life insurance and the premium expenditure of life insurance. Therefore, the regression results based on the IV model indicate that the higher the subjective life expectancy of middle-aged and older adult, the higher the participation rate of life insurance.

**Table 4:** Instrumental variable method regression results.

	(1)	(2)	(3)
Variables	SLE	IV-Probit	IV-Tobit
Parent	0.046***		
	(5.625)		
SLE		4.229**	63.708**
		(2.300)	(2.397)
Age	−0.000	−0.017***	−0.254***
	(−0.824)	(−4.164)	(−4.153)
Gender	0.028***	−0.191**	−2.766**
	(4.580)	(−2.506)	(−2.515)
Hukou	0.068***	−0.267*	−4.023*
	(8.381)	(−1.848)	(−1.930)
Edu	0.021***	0.147***	2.017***
	(4.702)	(2.785)	(2.646)
Spouse	0.018**	−0.078	−1.116
	(1.963)	(−0.855)	(−0.846)
PPI	0.007	0.128*	1.743*
	(0.971)	(1.780)	(1.686)
Nchild	−0.004	−0.046*	−0.637*
	(−1.563)	(−1.774)	(−1.722)
Asset	0.016***	0.020	0.224
	(7.897)	(0.600)	(0.460)
Loan	−0.010	0.075	0.998
	(−0.552)	(0.512)	(0.475)
Health	−0.075***	0.321**	4.818**
	(−26.736)	(2.280)	(2.369)
Constant	0.541***	−4.224***	−61.947***
	(14.557)	(−3.824)	(−3.840)
Observations	10,611	10,611	10,611
Wald test		5.85**	6.50**

Robust t-statistics in parentheses.

***p < 0.01, **p < 0.05, *p < 0.1.

### 4.4 Robustness tests

#### 4.4.1 Replacing the model

The Probit model is replaced with Logit model for robustness test. Table 5 shows that the effect of subjective life expectancy on the demand for life insurance is still positive and passes the test at the 1% significance level. Subjective life expectancy of middle-aged and older adult significantly contributes to the participation in life insurance. This finding is consistent with the previous analysis, indicating that the results of the previous analysis are robust.

**Table 5:** Logit model regression results.

	(1)	(2)	(3)	(4)
Variables	LI	LI	LI	LI
SLE	0.748***	0.445**	0.853***	0.545***
	(4.646)	(2.511)	(5.126)	(2.967)
Gender		−0.191*		−0.200*
		(−1.757)		(−1.796)
Hukou		−0.012		0.046
		(−0.089)		(0.309)
Edu		0.485***		0.434***
		(7.217)		(6.091)
Spouse		−0.006		0.041
		(−0.035)		(0.227)
PPI		0.367***		0.471***
		(2.591)		(3.204)
Nchild		−0.150***		−0.193***
		(−2.876)		(−3.439)
Asset		0.178***		0.201***
		(5.272)		(5.793)
Loan		0.040		0.079
		(0.150)		(0.289)
Health		0.043		0.055
		(0.838)		(1.040)
Constant	−3.641***	−3.552***	−4.629***	−4.360***
	(−34.315)	(−5.407)	(−5.244)	(−3.930)
Observations	10,611	10,611	10,611	10,611
Province FE	NO	NO	YES	YES
YEAR FE	NO	NO	YES	YES

z-statistics in parentheses.

***p < 0.01, **p < 0.05, *p < 0.1.

#### 4.4.2 Adding fixed effect

This section adds year fixed effects and region fixed effects to absorb the confounding factors of not changing over time and not changing with region in the regression, so as to alleviate the missing variable bias to some extent. The results (Table 6) are consistent with the previous benchmark regression symbols, and the coefficient size varies little, indicating that the estimation results in this paper are robust.

**Table 6:** Fixed effect regression results.

	(1)	(2)	(3)	(4)
Variables	LI	LI	Prem	Prem
SLE	0.388***	0.231***	0.228***	0.137***
	(0.076)	(0.085)	(0.042)	(0.044)
Age		−0.019***		−0.008***
		(0.004)		(0.002)
Gender		−0.070		−0.061**
		(0.051)		(0.027)
Hukou		0.029		0.022
		(0.069)		(0.038)
Edu		0.207***		0.160***
		(0.034)		(0.021)
Spouse		0.007		−0.001
		(0.080)		(0.039)
PPI		0.202***		0.113***
		(0.066)		(0.035)
Nchild		−0.079***		−0.031***
		(0.025)		(0.011)
Asset		0.094***		0.048***
		(0.016)		(0.009)
Loan		0.047		0.016
		(0.129)		(0.078)
Health		0.023		0.011
		(0.025)		(0.013)
Constant	−2.356***	−2.283***	−0.249	−0.362
	(0.363)	(0.498)	(0.288)	(0.331)
Observations	10,490	10,490	10,611	10,611
Province FE	YES	YES	YES	YES
YEAR FE	YES	YES	YES	YES

Standard errors in parentheses.

***p < 0.01, **p < 0.05, *p < 0.1.

#### 4.4.3 Reducing the sample size

Selecting 80% of the sample randomly for robustness testing, the effect of subjective life expectancy on life insurance participation remains positive and the coefficient does not change much in Table 7. The empirical analysis in the previous section passes the robustness test.

**Table 7:** Reduced sample regression results.

	(1)	(2)	(3)	(4)
Variables	LI	LI	Prem	Prem
SLE	0.331***	0.184**	0.195***	0.108**
	(4.037)	(1.995)	(4.305)	(2.287)
Gender		−0.079		−0.056*
		(−1.423)		(−1.864)
Hukou		0.035		0.017
		(0.490)		(0.418)
Edu		0.223***		0.167***
		(6.197)		(7.471)
Spouse		−0.005		−0.018
		(−0.055)		(−0.430)
PPI		0.148**		0.073**
		(2.087)		(1.982)
Nchild		−0.057**		−0.024**
		(−2.224)		(−2.030)
Asset		0.087***		0.044***
		(4.987)		(4.708)
Loan		0.102		0.051
		(0.771)		(0.619)
Health		0.017		0.007
		(0.631)		(0.510)
Constant	−1.969***	−1.898***	0.154***	0.088
	(−37.624)	(−5.685)	(5.682)	(0.504)
Observations	8,489	8,489	8,489	8,489

z-statistics in parentheses.

***p < 0.01, **p < 0.05, *p < 0.1.

### 4.5 Heterogeneity analysis

To test the H2 hypothesis, this section focuses on the impact of heterogeneity characteristics among middle-aged and older adult on the purchase behavior of life insurance. The life insurance participation of different groups is analyzed from the perspectives of gender, age, hukou, education level and household assets of the respondents.

#### 4.5.1 Gender heterogeneity

To verify whether there is gender heterogeneity in the effect of subjective life expectancy on life insurance purchases, the samples are divided into men and women for heterogeneity analysis. The results (Table 8) show that subjective life expectancy is significantly positively associated with men making life insurance purchase decisions and is significant at the 1% level. The effect is not significant in the female sample. There is gender heterogeneity, verifying the H2 hypothesis. The effect of subjective life expectancy on life insurance participation is more significant in the male sample. The reason for this may be that men tend to have more economic and family responsibilities in society, so they may pay more attention to their life safety and health status. They may want to buy life insurance to ensure that the family economy is not hit too hard in an unfortunate event. Therefore, the increase in subjective life expectancy may enhance their willingness to buy life insurance.

**Table 8:** Gender heterogeneity results.

	Probit		Tobit	
	(1)	(2)	(3)	(4)
Variables	Male	Female	Male	Female
SLE	0.318***	0.082	0.168***	0.076
	(2.725)	(0.719)	(2.769)	(1.251)
Age	−0.017***	−0.019***	−0.008***	−0.008***
	(−3.150)	(−3.501)	(−2.861)	(−2.996)
Hukou	−0.165*	0.153*	−0.089*	0.093*
	(−1.770)	(1.695)	(−1.791)	(1.735)
Edu	0.159***	0.321***	0.108***	0.302***
	(3.744)	(6.574)	(4.216)	(8.919)
Spouse	0.002	0.027	−0.009	−0.003
	(0.019)	(0.262)	(−0.138)	(−0.067)
PPI	0.074	0.237***	0.034	0.134***
	(0.838)	(2.591)	(0.703)	(2.857)
Nchild	−0.084***	−0.025	−0.031**	−0.011
	(−2.636)	(−0.771)	(−2.050)	(−0.691)
Asset	0.090***	0.090***	0.045***	0.047***
	(4.015)	(4.088)	(3.684)	(3.898)
Loan	−0.036	0.071	−0.040	0.030
	(−0.194)	(0.416)	(−0.359)	(0.278)
Health	0.060*	−0.023	0.022	−0.007
	(1.786)	(−0.675)	(1.216)	(−0.358)
Constant	−2.077***	−2.081***	0.087	−0.156
	(−4.782)	(−4.775)	(0.375)	(−0.683)
Observations	5,323	5,288	5,323	5,288

z-statistics in parentheses.

***p < 0.01, **p < 0.05, *p < 0.1.

#### 4.5.2 Age heterogeneity

In this paper, samples aged 60 and under are classified as middle-aged and those aged 60 and over as older adult. Table 9 indicates that subjective life expectancy has a more significant impact on the likelihood and extent of life insurance participation among middle-aged individuals. In the sample of older adult, the impact is not significant. There is age heterogeneity, verifying the H2 hypothesis. The reason may be that middle age is a critical period for long-term financial planning, including retirement savings and asset inheritance. Middle-aged people with a longer subjective life expectancy may be more focused on financial arrangements after retirement and therefore more inclined to purchase life insurance as part of financial planning.

**Table 9:** Age heterogeneity results.

	Probit		Tobit	
	(1)	(2)	(3)	(4)
Variables	Middle aged	Older adult	Middle aged	Older adult
SLE	0.242**	0.013	0.194***	0.024
	(2.484)	(0.087)	(2.633)	(0.586)
Hukou	0.033	−0.087	0.074	−0.040
	(0.428)	(−0.760)	(1.154)	(−1.179)
Edu	0.194***	0.320***	0.181***	0.158***
	(5.162)	(5.327)	(5.744)	(7.063)
Spouse	0.024	−0.053	0.011	−0.009
	(0.218)	(−0.475)	(0.133)	(−0.298)
PPI	0.184***	0.060	0.128**	0.023
	(2.656)	(0.401)	(2.555)	(0.586)
Nchild	−0.088***	−0.022	−0.061***	−0.008
	(−2.803)	(−0.684)	(−2.706)	(−0.948)
Asset	0.080***	0.091***	0.063***	0.021***
	(4.332)	(3.141)	(4.281)	(2.615)
Loan	0.017	0.013	−0.019	0.006
	(0.124)	(0.039)	(−0.172)	(0.064)
Health	0.019	0.009	0.015	0.001
	(0.680)	(0.189)	(0.721)	(0.102)
Constant	−2.777***	−3.359***	−0.545***	−0.266***
	(−11.116)	(−8.953)	(−2.923)	(−2.632)
Observations	5,627	4,984	5,627	4,984

z-statistics in parentheses.

***p < 0.01, **p < 0.05, *p < 0.1.

#### 4.5.3 Urban–rural heterogeneity

This paper uses the location of the hukou to distinguish between urban and rural areas. From Table 10, it can be found that subjective life expectancy is significantly and positively associated with life insurance decisions made by urban middle-aged and older adult, and is significant at the 1% level. There is urban–rural heterogeneity, verifying the H2 hypothesis. The reason may be that the insurance market in the city is relatively developed and mature, which provides diversified insurance products and services. This market environment enables middle-aged and older adult to choose the right insurance products according to their own needs and preferences, thus increasing the possibility that they can buy life insurance.

**Table 10:** Urban–rural heterogeneity results.

	Probit		Tobit	
	(1)	(2)	(3)	(4)
Variables	Urban	Rural	Urban	Rural
SLE	0.508***	0.088	0.376***	0.048
	(2.863)	(0.951)	(3.154)	(1.048)
Age	−0.012*	−0.020***	−0.009*	−0.007***
	(−1.766)	(−4.285)	(−1.856)	(−3.569)
Gender	−0.365***	0.013	−0.275***	−0.008
	(−3.458)	(0.226)	(−3.654)	(−0.287)
Edu	0.189***	0.246***	0.153***	0.192***
	(3.574)	(6.088)	(3.962)	(7.674)
Spouse	−0.020	0.036	−0.043	0.007
	(−0.142)	(0.372)	(−0.435)	(0.169)
PPI	0.333**	0.125*	0.292***	0.052
	(2.256)	(1.780)	(3.037)	(1.471)
Nchild	−0.126**	−0.042*	−0.070**	−0.014
	(−2.401)	(−1.659)	(−2.220)	(−1.209)
Asset	0.039	0.107***	0.022	0.053***
	(1.342)	(5.751)	(1.063)	(5.613)
Loan	0.067	0.012	0.125	−0.045
	(0.314)	(0.077)	(0.671)	(−0.532)
Health	0.086*	0.001	0.066*	−0.004
	(1.690)	(0.042)	(1.884)	(−0.306)
Constant	−2.038***	−2.077***	0.098	−0.038
	(−3.582)	(−5.653)	(0.251)	(−0.214)
Observations	2,043	8,568	2,043	8,568

z-statistics in parentheses.

***p < 0.01, **p < 0.05, *p < 0.1.

#### 4.5.4 Educational heterogeneity

Highly educated people usually have stronger risk perception and insurance awareness. They are able to assess risks and make appropriate protection decisions rationally. With the increase of subjective life expectancy, they pay more attention to future problems such as bequest motive and medical care, thus increasing the demand for life insurance. In the section, the samples are grouped according to education level, with those who have only completed primary school or below classified as the low education group, and those with a junior high school diploma or higher as the high education group. Table 11 shows that in the high-education group, life insurance participation rate grows as the subjective life expectancy increases. While in the low education group, subjective life expectancy has no significant effect on life insurance participation rate. The heterogeneity hypothesis H2 is proved again.

**Table 11:** Educational heterogeneity results.

	Probit		Tobit	
	(1)	(2)	(3)	(4)
Variables	Low-edu	High-edu	Low-edu	High-edu
SLE	0.008	0.433***	0.016	0.406***
	(0.074)	(3.578)	(0.391)	(3.764)
Age	−0.022***	−0.011*	−0.007***	−0.010**
	(−4.173)	(−1.946)	(−4.012)	(−2.065)
Gender	0.098	−0.272***	0.034	−0.262***
	(1.423)	(−3.803)	(1.334)	(−3.868)
Hukou	0.018	−0.011	0.025	−0.007
	(0.173)	(−0.142)	(0.659)	(−0.098)
Spouse	0.048	−0.130	0.016	−0.146
	(0.460)	(−1.052)	(0.482)	(−1.293)
PPI	0.050	0.274***	0.013	0.222***
	(0.576)	(2.973)	(0.402)	(2.883)
Nchild	−0.052*	−0.052	−0.016*	−0.031
	(−1.775)	(−1.418)	(−1.706)	(−1.000)
Asset	0.085***	0.092***	0.028***	0.087***
	(3.854)	(4.128)	(3.511)	(4.193)
Loan	0.099	−0.031	0.017	−0.039
	(0.559)	(−0.173)	(0.219)	(−0.228)
Health	−0.021	0.056	−0.012	0.051
	(−0.662)	(1.617)	(−0.973)	(1.636)
Constant	−1.360***	−1.964***	0.398***	0.088
	(−3.327)	(−4.671)	(2.699)	(0.236)
Observations	7,195	3,416	7,195	3,416

z-statistics in parentheses.

***p < 0.01, **p < 0.05, *p < 0.1.

#### 4.5.5 Heterogeneity of household wealth

The paper selects the median of household wealth as the dividing line between low-asset households and high-asset households. Table 12 shows that the effect of subjective life expectancy on life-insurance participation is more significant in the high-asset sample. There is wealth heterogeneity in the impact of subjective life expectancy on life insurance demand, verifying the hypothesis of H2. People with high assets usually have higher financial freedom, and do not worry too much about their daily expenses and basic living expenses. Therefore, they are capable and willing to consider future financial planning and risk management. For middle-aged and older people, they may focus more on long-term financial security and heritage planning as subjective life expectancy increases. Life insurance, as a financial tool, can naturally provide long-term protection and wealth inheritance for middle-aged and older people with high assets.

**Table 12:** Wealth heterogeneity results.

	Probit		Tobit	
	(1)	(2)	(3)	(4)
Variables	Low-asset	High-asset	Low-asset	High-asset
SLE	0.142	0.263**	0.069	0.193***
	(1.153)	(2.467)	(1.289)	(2.826)
Age	−0.028***	−0.015***	−0.010***	−0.009***
	(−4.808)	(−3.100)	(−4.446)	(−2.844)
Gender	0.000	−0.128**	−0.004	−0.111***
	(0.005)	(−1.977)	(−0.108)	(−2.583)
Hukou	0.012	0.045	0.004	0.034
	(0.123)	(0.536)	(0.094)	(0.595)
Edu	0.287***	0.212***	0.194***	0.180***
	(5.790)	(5.128)	(7.024)	(6.017)
Spouse	0.038	−0.009	−0.009	0.004
	(0.343)	(−0.080)	(−0.201)	(0.053)
PPI	0.234**	0.119	0.116***	0.072
	(2.363)	(1.460)	(2.729)	(1.363)
Nchild	−0.073**	−0.046	−0.027**	−0.020
	(−2.230)	(−1.464)	(−2.163)	(−1.107)
Loan	0.036	0.052	−0.007	0.029
	(0.164)	(0.341)	(−0.067)	(0.258)
Health	0.001	0.014	−0.001	0.007
	(0.041)	(0.450)	(−0.059)	(0.338)
Constant	−0.778**	−1.264***	0.555***	0.469**
	(−2.019)	(−3.773)	(3.274)	(2.214)
Observations	5,334	5,277	5,334	5,277

z-statistics in parentheses.

***p < 0.01, **p < 0.05, *p < 0.1.

## 5 Conclusion

Focusing on the aging of the population and risk management, this paper aims to understand the changes in life insurance purchase. Using Probit model and Tobit model over data from 2011 to 2018 in CHARLS, this paper investigates the impact of subjective life expectancy on life insurance participation among middle-aged and older adult from a micro point of view, providing a new perspective to explain life insurance participation in China. The empirical results show that subjective life expectancy positively influences life insurance purchase decision-making and proportion of expenditure among middle-aged and older adults. Considering the possible endogeneity as well as the robustness of subjective life expectancy, we replace the model and change the sample size. The findings remain robust. The control variables, such as age, education, number of children, social security and total household assets have significantly different influence on life insurance participation. This study will emphasize helping consumers, especially middle-aged and older people, to better assess their own insurance needs and make more informed purchase choices.

Middle-aged and older adult’ subjective life expectancy exhibits significant heterogeneity in its impact on the likelihood and extent of their participation in life insurance. Specifically, subjective life expectancy significantly influences men’s participation in life insurance, while its impact on women’s participation is not significant. The subjective life expectancy of middle-aged individuals significantly affects their participation in life insurance, whereas its impact on the older adult is not significant. Furthermore, urban–rural differences also significantly affect the life insurance participation of the middle-aged and older adult. An increase in subjective life expectancy significantly boosts the life insurance participation rate among urban residents. In terms of educational heterogeneity, respondents with higher education significantly increase the proportion of life insurance in their asset allocation as their subjective life expectancy increases. In contrast, respondents with lower education, due to lower risk awareness, also have a relatively lower rate of participation in life insurance. Lastly, when examining different wealth groups, we find that as subjective life expectancy increases, the life insurance participation rate of high-asset families shows a significant upward trend, while the life insurance participation rate of low-asset families does not change much. These findings provide valuable insights for a deeper understanding of the factors influencing the participation of the middle-aged and older adult in life insurance.

## 6 Discussion

We suggest several directions for future research, and part of the work has been doing. First, optimistic or pessimistic subjective life expectation can lead to behavioral decisions distortion, such as life insurance purchases (55–57). The emotion brought by the comparison of subjective life expectancy and chronological age should be further tested to explain the effect from subjective life expectancy to purchasing life insurance. For example, the older who is pessimistic subjective life expectation has anxious feeling, and influence decision and behavior (58–60, 70). In addition, we find in the current study that as people age, their emotion of subjective life expectation may swing from pure pessimistic to really optimistic. Further study will apply financial mathematical methods and solve time inconsistencies.

Second, alternative methods need to be used because a single method may not be sufficient to explain human decision-making. We employed behavioral economics and prospect theory to deal with the relationship between subjective life expectancy and life insurance participation (61–64). However, how to define reference point about lifespan is the core of the difficulty.

Third, further research on the determinants of subjective life expectancy will enrich the understanding of the relationship between subjective life expectancy and life insurance purchase. These research may include quantitative studies examining the correlation between subjective life expectancy and insurance purchasing behavior, as well as qualitative studies exploring the underlying psychological mechanisms and cultural factors that influence this relationship. Such studies would not only help us to understand more deeply the psychological basis of life insurance demand, but also have practical implications for the insurance industry in terms of product design, marketing strategies and risk management.

Last but not least, we suggest that future studies could further explore the relationship between the different types of life insurance products (including annuities) and subjective life expectancy. This will facilitate a more comprehensive understanding of people’s insurance needs and purchasing behaviors at different life-cycle stages.

## Data Availability

The datasets presented in this study can be found in online repositories. The names of the repository/repositories and accession number(s) can be found in the article/supplementary material.
